# Body Composition and Biochemical Parameters of Nutritional Status: Correlation with Health-Related Quality of Life in Patients with Colorectal Cancer

**DOI:** 10.3390/nu12072110

**Published:** 2020-07-16

**Authors:** Luz-Ma-Adriana Balderas-Peña, Faviola González-Barba, Brenda-Eugenia Martínez-Herrera, Ulises-Rodrigo Palomares-Chacón, Oscar Durán-Anguiano, Mario Salazar-Páramo, Eduardo Gómez-Sánchez, Carlos Dávalos-Cobián, Arnulfo-Hernán Nava-Zavala, Guillermo-Allan Hernández-Chávez, Daniel Sat-Muñoz

**Affiliations:** 1 Unidad de Investigación Biomédica 02, UMAE Hospital de Especialidades (HE), Centro Médico Nacional de Occidente (CMNO), Instituto Mexicano del Seguro Social (IMSS), 1000 Belisario Domínguez, Colonia Independencia, Guadalajara, Jalisco 44340, Mexico; lmabp@yahoo.com.mx (L.-M.-A.B.-P.); faviolagonzalez1992@gmail.com (F.G.-B.); bren.mtzh16@gmail.com (B.-E.M.-H.); navazava@yahoo.com.mx (A.-H.N.-Z.); 2 Departamento de Morfología, Centro Universitario de Ciencias de la Salud (CUCS), Universidad de Guadalajara (UdG), Cuerpo Académico UDG CA-874 “Ciencias morfológicas en el diagnóstico y tratamiento de la enfermedad”, 950 Sierra Mojada, Gate 7, Building C, 1st level, Colonia Independencia, Guadalajara, Jalisco 44340, Mexico; 3 Hospital General de Zona (HGZ) #02 c/MF “Dr. Francisco Padrón Puyou”, Delegación Estatal San Luis Potosí, IMSS, Benigno Arriaga street and Melchor Ocampo S/N, Colonia Tequisquiapan, San Luis Potosí, San Luis Potosí 78250, Mexico; 4 Servicio de Coloproctología, Unidad Médica de Alta Especialidad (UMAE), Hospital de Especialidades (HE), Centro Médico Nacional de Occidente (CMNO), Instituto Mexicano del Seguro Social (IMSS), 1000 Belisario Domínguez, Colonia Independencia, Guadalajara, Jalisco 44340, Mexico; ulisespalomares@gmail.com (U.-R.P.-C.); droscardurananguiano@gmail.com (O.D.-A.); 5 Academia de Inmunología, Departamento de Fisiología, Centro Universitario de Ciencias de la Salud (CUCS), Universidad de Guadalajara (UdG), 950 Sierra Mojada, Gate 7, Building O, 1st level, Colonia Independencia, Guadalajara, Jalisco 44340, Mexico; msalazpa@gmail.com; 6 División de Disciplinas Básicas para Salud, Centro Universitario de Ciencias de la Salud (CUCS), Universidad de Guadalajara (UdG), Sierra Mojada 950, Edificio N, Puerta 1, Planta Baja, Colonia Independencia, Guadalajara, Jalisco 44340, Mexico; eduardo.gsanchez@academicos.udg.mx; 7 Departamento Clínico de Gastroenterología Endoscópica, Unidad Médica de Alta Especialidad (UMAE), Hospital de Especialidades (HE), Centro Médico Nacional de Occidente (CMNO,) Instituto Mexicano del Seguro Social (IMSS), 1000 Belisario Domínguez, Colonia Independencia, Guadalajara, Jalisco 44340, Mexico; endosmedica@gmail.com; 8 Programa Internacional Facultad de Medicina, Universidad Autónoma de Guadalajara, Av. Patria 1201, Lomas del Valle, Zapopan 45129, Mexico; 9 Servicio de Inmunología y Reumatología, División de Medicina Interna, Hospital General de Occidente, Secretaria de Salud Jalisco, Av. Zoquipan 1050, Zoquipan, Zapopan, Jalisco 45170, Mexico; 10 División de Oncología Hematología, UMAE, Hospital de Especialidades, Centro Médico Nacional de Occidente, Instituto Mexicano del Seguro Social, 1000 Belisario Domínguez, Colonia Independencia, Guadalajara, Jalisco 44340, Mexico; memin075@gmail.com; 11 Departamento Clínico de Oncología Quirúrgica, División de Oncología Hematología, UMAE, Hospital de Especialidades, Centro Médico Nacional de Occidente, Instituto Mexicano del Seguro Social, 1000 Belisario Domínguez, Colonia Independencia, Guadalajara, Jalisco 44340, Mexico

**Keywords:** nutritional status, body composition, colorectal neoplasms, quality of life, patient-reported outcome measures

## Abstract

Up to 60% of colorectal cancer (CRC) patients develop malnutrition, affecting treatment effectiveness, increasing toxicity, postoperative complications, hospital stay, and worsening health-related quality of life (HRQOL). This cross-sectional study analyzed data from 48 women and 65 men with CRC. We correlated scores of the scales from the questionnaires EORTC (European Organisation for Research and Treatment of Cancer) Quality of Life Questionnaire Core 30 (QLQ)-C30 and Colorectal Cancer module Colorectal 29 (QLQ-CR29) with patients’ body composition and clinical and biochemical indicators of nutritional status. Results: Scores on quality of life were negatively associated with the lymphocyte count (rP = −0.386) and the fat trunk percentage (rP = −0.349) in the women’s group. Scores on the physical and role functioning were inversely associated with the adiposity percentage (rP = −0.486 and rP = −0.411, respectively). In men, total skeletal muscle mass (SMM) was positively associated with emotional functioning (rP = 0.450); the trunk SMM was negatively related to fatigue (rP = −0.586), nausea and vomiting (rP = −0.469), pain (rP = −0.506), and financial difficulties (rP = −0.475); additionally, serum albumin was positively related to physical, emotional, and social functioning scales (rPs = 0.395, 0.453, and 0.363, respectively) and negatively to fatigue (rP = −0.362), nausea and vomiting (rP = −0.387), and appetite loss (rP = −0.347). Among the men, the reduced SMM and biochemical, nutritional parameters were related to low scores on the EORTC QLQ-C30 and QLQ-CR29 functioning scales. In conclusion, in patients with CRC, malnourishment could have a profound effect on the patients’ functionality and QoL (quality of life).

## 1. Introduction

Colorectal cancer (CRC) is considered a global disease; its annual incidence is approximately about 1,000,000 new cases. It is the fourth most common type of cancer around the world and the second leading cause of death in the United States [1]. CRC is the third most common type of cancer in men (10.0%) and the second most common in women (9.4%) worldwide. Nearly 60% of cases occur in developed countries. In Latin America, it is the third most common type of cancer [2].

Weight loss is an important prognostic factor in oncologic patients [3]. Between 30% and 80% of them lose weight [3,4]. The presence of exacerbated proteolysis and lipolysis, in addition to the reduced protein synthesis triggered by the tumor-associated cachexia [3,5] generate a catabolic profile characterized by the loss of muscle mass and fat. This leads to physiological and functional alterations, affecting the patients’ physical, psychological, and social status and their daily performance of the activities of daily living [6,7].

Among patients with CRC and weight loss, the frequency of malnutrition is approximately 30% to 60% [3,4,8]. The metabolic environment and the treatment modalities (combinations of chemotherapy, radiotherapy, and surgery) can cause acute and chronic symptoms that limit feeding and affect the nutritional status [3,4,8], which leads to morbidity and increased mortality in patients with the malignant digestive disease [5,6,9,10,11,12,13].

The literature describes different nutritional assessment methods [3,14,15], including subjective global assessment, body composition analysis, and measurement of biochemical markers (total proteins, albumin, prealbumin, lymphocyte count, and transferrin, among others). The albumin level is one of the parameters most frequently used [3,16], and its response to nutritional treatment is an excellent prognostic factor for cancer survival [16].

Previous studies have demonstrated a negative association between nutritional status and health-related quality of life (HRQOL) [6,7,17,18]. Deterioration in nutritional status influences the prognosis and therapeutic compliance, and treatment effectiveness increases the risk of toxicity and postoperative complications and reduces the HRQOL [8,9,10,11]. The aim of this study was to determine the relation of HRQOL to total protein, albumin levels, other biochemical data, and body composition compounds in patients with CRC.

## 2. Material and Methods

### 2.1. Study Design

This was a cross-sectional study, with a retrospective collection of laboratory data.

### 2.2. Patients

From 2016 to 2017, one-hundred thirteen patients > 18 years old with CRC who were treated in the Coloproctology, Surgical Oncology, and Medical Oncology Clinical Departments from Unidad Medica de Alta Especialidad, Hospital de Especialidades, Centro Médico Nacional de Occidente, Instituto Mexicano del Seguro Social at Guadalajara, Mexico, were recruited.

### 2.3. Collection of Information

Informed consent was obtained from all individual participants included in the study. After the patients signed their informed consent forms, their nutritional status and HRQOL were evaluated. The height was measured with the Seca 213 stadiometer (Seca, Hamburg, Germany). A bioelectrical impedance analysis device, the BF-601F FitScan Segmental Body Composition Monitor (Tanita, Tokyo, Japan) was used to assess weight, body mass index (BMI), total percentage of body fat, total skeletal muscle mass (SMM), and segmental skeletal muscle mass. The sum of the muscle masses of the four limbs (appendicular muscle mass) was divided by the height (in meters) squared to calculate the skeletal muscle index.

Using the technique recommended by the American Society of Hand Therapists, we measured handgrip strength (HGS) three times for each hand with a one-minute recovery period between them and recorded the maximum result.

The laboratory measurements were conducted as part of the clinical routine and then abstracted from medical charts, including the levels of the biochemical indicators of nutritional status (cholesterol, total proteins, albumin, and total lymphocyte count).

The HRQOL was evaluated with the validated European Organisation for Research of Cancer Quality of Life (EORTC) Quality of Life Questionnaire (QLQ)-C30 and QLQ-CR29 questionnaires (validated Mexican Spanish Version 3) [19,20,21].

The patients’ sociodemographic data, information about the clinical stage and anatomical location of the tumor, and information about treatment modality were obtained from the clinical charts.

### 2.4. Calculation of Health-Related Quality of Life and Satisfaction Questionnaires’ Scores

The questionnaires contained one-item and multi-item scales [22]. The EORTC QLQ-C30 questionnaire includes five functional multi-item scales (physical, role-based, cognitive, emotional, and social functioning), three symptoms scales (fatigue, pain, and nausea-vomiting), one scale for global health status/quality of life (GHS/QoL), and six single-item scales. Each multi-item scale includes a different set of items, and no item is involved in more than one scale. All the scales have a similar structure.

The scoring method for every scale is the same as follows: (1) to calculate the items’ average (of each item that contributes to building the scale) and obtain the raw score; and (2) to apply the linear transformation formula to standardize the raw score and convert it to a scoring system from 0 to 100 according to the instructions provided in the EORTC QLQ-C30 Scoring Manual [22]. A higher score on a functional or global status scale represents a higher level of function or a better HRQOL, respectively. A high score on the symptom scales represents increased severity of symptoms and, thus, the presence of more health problems and worse HRQOL [23].

For the EORTC QLQ-CR29, specific module scoring was used in the same procedure previously described. This module includes five functional scales (body self-image, anxiety, weight, interest in sexual contact in males and females) and 18 scales of signs and symptoms (urinary frequency, blood and mucus in stool, stool frequency, urinary incontinence, dysuria, abdominal pain, buttock pain, bloating, dry mouth, hair loss, taste, flatulence, fecal incontinency, sore skin, embarrassment, stoma care problems, impotence, and dyspareunia) [20,22].

### 2.5. Ethical Considerations

All procedures performed in studies involving human participants were in accordance with the ethical standards of the institutional and/or national research committee and with the 1964 Helsinki Declaration and its later amendments or comparable ethical standards. The study was approved by the Institutional Review Board (Comisión Nacional de Investigación Científica of Instituto Mexicano del Seguro Social) (Institutional Review Board Registration No. 2013-785-073).

### 2.6. Statistical Analysis

Quantitative data are reported as the means ± standard deviations or as medians with interquartile intervals. To describe the qualitative data, we use percentages and proportions. We identified differences between the genders with Student’s *t*-test. The association was estimated with Pearson’s correlation coefficient. A *p*-value of less than 0.05 was considered significant. All data were processed in the two following statistical packages: Excel 2010^®^ (Microsoft Corporation, Redmond, WA) and SPSS^®^ Version 16 statistical software (SPSS Inc, Chicago, IL, USA).

## 3. Results

### 3.1. Characteristics of the Population.

We analyzed data from 113 patients with CRC, 48 women (42.48%) and 65 men (57.52%). By clinical-stage, ten patients (8.8%) had clinical stage I disease, 41 (36.3%) had clinical stage II disease, 38 (33.6%) had clinical stage III disease, and 16 (14.2%) had clinical stage IV disease. In eight patients, the clinical stage could not be classified (data were incomplete because the patients had begun treatment in another health institution).

### 3.2. Nutritional Status

Women and men had significant differences in mean weight (65.14 and 74.6 kg, respectively) and total fat percentage (33.75% and 28.05%, respectively). The HGS was higher in men. With regard to the biochemical indicators or nutritional status, we found higher levels of cholesterol in women than in men (see Table 1).

### 3.3. Health-Related Quality of Life

The HRQOL analysis of the EORTC QLQ-C30 questionnaire scores yielded the following results: For the multi-item scales, Cronbach’s α > 0.7, except for cognitive functioning (Cronbach α = 0.456). The analysis by gender of the GHS/QoL scale scores showed scores higher than 75 points in both sexes (non-significant). Emotional and social functioning scale scores were significantly different between the sexes (*p* < 0.05). The symptom scale with the highest scores was that for financial difficulties (score range: 28 to 30), followed by fatigue, insomnia, constipation, and pain (scores higher than 20; see Table 2).

For the EORTC QLQ-CR29 multi-item scales, Cronbach’s α reached 0.815. Functioning scale scores were not significantly different in the gender analysis, but the women’s scores were, on average, approximately 10 points lower than the men’s score. On the symptom scales, women’s scores for stool frequency, abdominal pain, bloating, and hair loss were higher than men’s scores (*p* < 0.05; see Table 2).

### 3.4. Relation between Health-Related Quality of Life and Nutritional Status in Women with Colorectal Cancer

In the correlation analysis between scores on the EORTC QLQ-C30 questionnaire scales and body composition in women, we found a negative correlation between GHS/QoL scores and trunk fat in women (rP = −0.349; *p* = 0.05). HGS was positively correlated with role functioning (rP = 0.399; *p* = 0.024). We also found a positive correlation between total body fat percentage and scores on the dyspnea and insomnia symptom scales (*p* < 0.05).

In the EORTC QLQ-CR29 questionnaire scores, we found the following significant correlations with the anthropometric nutritional indicators: BMI was positively associated with scores on the fecal incontinence symptom scale (rP = 0.305; *p* = 0.044), and scores on the dry mouth and embarrassment scales were negatively related to the percentage of trunk fat (see Table 3).

With regard to biochemical, nutritional parameters, the number of lymphocytes was positively correlated with scores on the GHS/QoL, and the total cholesterol level was positively associated both with scores on the weight functional scale of the EORTC QLQ-CR29 questionnaire and with scores on the symptom scale for blood and mucus in the stool of the same questionnaire (see Table 3).

### 3.5. The Relation between Health-Related Quality of Life and Nutritional Status in Men with Colorectal Cancer

Scores on the GHS/QoL scale were negatively related to the percentage of trunk fat (rP = −0.341; *p* = 0.045). SMM was positively related to emotional functioning. The SMM of the trunk was positively related to scores on the physical, role, emotional, and social functioning scales of the EORTC QLQ-C30 questionnaire and negatively related to scores on the fatigue, nausea and vomiting, pain, and financial difficulties symptom scales. With regard to the EORTC QLQ-CR29 module, the SMM of the trunk was positively associated with scores on the body image, anxiety, and weight functional scales and negatively associated with scores on the urinary frequency and taste symptom scales (see Table 3).

HGS had a positive relation to scores on the physical, emotional, and social functioning scales, and it was negatively associated with fatigue, constipation, urinary incontinence, flatulence, and embarrassment (*p* < 0.05; see Table 3).

The serum albumin levels were positively related to scores on the physical, emotional, and social functioning scales and negatively related to scores on fatigue, nausea, and vomiting, appetite loss, constipation, diarrhea, body image, and weight (*p* < 0.05; see Table 3).

## 4. Discussion

Malnutrition can importantly affect overall treatment and QoL in CRC patients. In this cross-sectional study, we studied 113 patients with CRC, assessing clinical and biochemical nutritional indicators, and correlating with QoL parameters. We found several differences among our patients when different biomarkers were correlated with QoL parameters.

Around the world, the rate of mortality from CRC has decreased as part of an epidemiological transition of the disease from a condition with high lethality rates to a high-cost chronic illness with growing numbers of survivors. This transition is a result of earlier diagnosis and improved surgical, medical, and radiotherapy treatments [19].

As a result, the needs of patients from their point of view must be addressed during treatment and afterward [23] because HRQOL strongly affects clinical outcomes (recurrence or survival). Thus, the HRQOL results can guide patients and doctors in their choice of treatment options and in making an informed decision about treatment [20]. Over the past 20 years, the American Institute for Cancer Research and the World Cancer Research Fund have accordingly emphasized the effect of nutrition on patients with cancer [19] because approximately 30% to 60% of patients with CRC develop malnutrition [3,4].

In our patients, we observed low levels of cholesterol and lymphocytes, which are considered indicators of mild malnourishment. As other authors mentioned, our findings may represent the combined effect of the treatment modalities on the rapid growth of cells (such as immune cells) and high rates of cell destruction; the consequence is increased needs for lipoproteins (such as cholesterol) for cell membrane synthesis [22,23,24].

In contrast to our opinion, other authors believe that pronounced weight loss rate and malnourishment indicators observed in patients with gastrointestinal malignancies are related to appetite loss and anorexia. Different measurement approaches on QoL and albumin in the present and other studies may explain the observed differences between studies [25,26,27].

The high serum albumin levels, SMM, SMM of the trunk, skeletal muscle index, and muscle functioning (evaluated through HGS) in our male patients were associated with high scores on the function scales. Such conditions positively affect performance and HRQOL in men with CRC.

In all our patients, the albumin levels were higher than 4.18 g/dL, whereas other authors [24,28] have found that 49% of patients have albumin levels below 3.5 g/dL, some even lower than 2.8 g/dL. Because of these data [28], the link between albumin level and HRQOL is unclear.

In previous reports, a 10% reduction in the BMI was predictive of poorer HRQOL [29,30], and other authors reported that high BMI is correlated with high scores on HRQOL scales in different questionnaires and with self-perceived better appearance [31,32]. Conversely, in our study, we did not observe this BMI behavior pattern; however, HRQOL was directly related to muscle mass quantity, protein levels, and albumin serum levels, more so than was BMI.

Our data revealed that muscle mass loss is directly related to fatigue and weakness [33,34], both of which compromise function and HRQOL in oncologic patients.

Neefjes et al. studied 233 patients with advanced cancer who were treated with palliative chemotherapy alternatives for different kinds of cancer, including CRC [35]; they calculated the skeletal muscle mass index SMMI and studied its relationship with fatigue through the Functional Assessment of Chronic Illness Therapy (FACIT) questionnaire (with the scale cancer-related fatigue) [34]. They described an inverse association between cancer-related fatigue and SMMI in men (beta = −0.447; *p* = 0.004), but not in women (beta = 0.401; *p* = 0.090); these findings were similar to the results of our analysis of fatigue and other symptoms through the EORTC QLQ-C30 and EORTC QLQ-CR29 questionnaires.

Even though Neefjes et al. [36] did not use the same statistical methods, we believe that work with the FACIT-fatigue questionnaire yielded convincing evidence about the relationship between muscle mass and higher scores on fatigue symptom scales and that it supports our current results with the EORTC QLQ-C30 fatigue scale.

Different authors [34,35] have described a significant loss of muscle mass in male patients with CRC, a situation that can be linked to poor performance. This situation was present in our population, inasmuch as we observed a direct relation between the SMM of the trunk and physical functioning, the latter of which was also linked to the HGS. We consider this evidence that HGS is an important indicator of nutritional status.

Feather et al. described the adverse effect of oxaliplatin treatment inducing muscle mass loss [33]. In our study, more than 50% of the patients were receiving a chemotherapy regimen that included oxaliplatin; this situation is associated with the development of cachexia and could affect SMM, particularly in the women we studied.

These data support the hypothesis proposed by van der Werf et al. [37] in their published protocol about muscle mass loss and treatment failure in patients with metastatic CRC. Because chemotherapy has an unfavorable effect on muscle mass and sufficient protein intake and physical activity have a combined positive effect on the induction of muscle protein anabolism in patients with metastatic CRC, they argued that during treatment with first-line chemotherapy, preserving muscle mass may improve clinical and patient-reported HRQOL [37].

Van der Werf et al. proposed the use of clinical and patient-reported outcomes, similar to those used in our study: skeletal muscle area, quality of life (EORTC QLQ-C30), HGS, treatment toxicity, treatment intensity, adverse events, treatment response, and progression-free and overall survival [38]. We think that our results can provide valuable information to other groups in choosing parameters to study.

Our female patients with CRC had diminished muscle mass and reduced HGS in comparison with the male patients. This situation is, in many ways, a typical difference between genders; however, it is not normal to find such profound muscle mass loss exclusively in women with CRC [38].

We observed that at least 75% of the women and 50% of the men had diminished HGS. With regard to clinical stage, seventy-five percent of the women with clinical stage II and III disease had an HGS of less than 20 kg, and fifty percent of the men in all clinical stages had an HGS of less than 30 kg.

The present study displayed several strengths as the use of contemporary and validated measures of QoL, the use of various methods for measuring body composition and hand grip, and even more, the use of routine available biomarkers.

However, we acknowledge limitations like the sample size that impaired us from reaching a more sophisticated search and adjusting the statistical analysis for confounders. 

Other disadvantages of our design were: (1) the retrospective collection of laboratory data and (2) the lack of CT scan screening for body composition evaluation.

Although BIA (bioimpedance analysis) is a better screening tool, to achieve lower sensitivity and specificity, compared to CT scan or DXA (dual-energy X-ray absorptiometry), both are considered gold standards in body composition evaluation.

Furthermore, the cross-sectional design we performed involved only association tests, and no calculation of relative risks was allowed. Nevertheless, our findings give major clues that should guide the focus of future studies.

In patients with cancer who have marginal gastrointestinal function, intensive nutrition support (enteral or parenteral) is an indicated treatment. Using the EORTC QLQ-C30, Karnofsky Performance Status questionnaire, and Subjective Global Assessment, Vashi et al. examined the effect of parenteral nutrition on HRQOL and nutritional outcomes in patients with different types of advanced cancer who were receiving home support in the form of parenteral nutrition [34,35]. They documented an improvement in scores on various function and symptom scales of the EORTC QLQ-C30 (the scales for GHS/QoL, physical functioning, role functioning, and emotional functioning and the symptom scales for fatigue, nausea and vomiting, appetite loss, and constipation). These results represent strong evidence about the nutritional state’s positive influence on HRQOL and functionality for patients with different types of cancer [36].

## 5. Conclusions

In summary, patients with CRC have impaired functioning as a direct result of the disease, its treatment, and the disease progression, but malnourishment could have a profound effect on the patients’ functionality and QoL. In these conditions, nutritional assessment and early treatment to prevent malnutrition or cancer-related cachexia and to stop muscle mass loss, especially in women with CRC, could enhance patients’ functionality and HRQOL.

## Figures and Tables

**Table 1:** Comparison by sex of the anthropometrical, biochemical, and functional indicators of nutritional status.

Variable	Female		Male		*p* *
	Mean	(SD)	Mean	(SD)	
**Anthropometric Characteristics**					
Age (years)	56.40	(±12.95)	64.25	(±11.04)	*0.001*
Weight (kg)	65.14	(±14.96)	74.61	(±13)	*0.000*
Height (cm)	160.7	(±8.24)	169	(±7.9)	*0.000*
Weight at diagnosis date (kg)	66.94	(±10.04)	65.18	(±23.25)	0.746
BMI (kg/m^2^)	25.29	(±5.49)	25.43	(±5.63)	0.896
Total Body Fat (%)	33.75	(±6.78)	28.05	(±9.79)	*0.008*
**Skeletal Muscle Mass by Body Segments**					
SMM (kg)	40.67	(±6.55)	50.53	(±7.43)	0.811
Right arm	2.59	(±2.66)	3.61	(±4.27)	0.593
Trunk	23.19	(±3.39)	27.61	(±6.30)	0.108
SMI (kg/m^2^)	6.94	(±1.98)	8.71	(±5.19)	0.074
Handgrip strength (kg)	15.54	(±5.27)	27.24	(±9.19)	*0.000*
**Biochemical Markers of Nutritional Status**					
Total Protein	7.48	(±0.57)	7.23	(±0.78)	0.145
Albumin	4.18	(±0.53)	4.17	(±0.85)	0.942
TLC (cells/mm^3^)	1725.18	(±721.17)	1525.51	(±728.15)	0.238
Cholesterol	182.92	(±24.92)	165.40	(±33.74)	*0.022*
**Tumor Location, Clinical, and Treatment Characteristics**					
	*n* = 48	(percentage)	*n* = 65	(percentage)	*p*-value
**Anatomic tumor location**					
1. Colon	14	(29.2%)	19	(29.2%)	
2. Rectum	34	(70.9%)	46	(70.8%)	0.582
**Clinical Stage**					
Non-classifiable	2	(4.2%)	1	(1.5%)	
I	3	(6.3%)	7	(10.8%)	
II	19	(39.6%)	22	(33.8%)	
III	19	(39.6%)	24	(36.9%)	
IV	5	(10.4%)	11	(16.9%)	0.648
**Stoma**					
1. Yes	30	(62.5%)	47	(72.3%)	
2. No	18	(37.5%)	18	(27.7%)	0.183
**Chemotherapy**					
Neo-adjuvant	23	(47.9%)	22	(33.8%)	
Adjuvant	10	(20.8%)	17	(26.2%)	
Palliative	3	(6.3%)	4	(6.2%)	
Adjuvant palliative	0	(0%)	4	(6.2%)	
Not received	12	(25%)	18	(27.7%)	0.300

* Significance level: *p* < 0.05. Statistics: Student *t*-test for numerical values, Fisher exact test for proportion and/or percentage values; SD = standard deviation; BMI = body mass index; SMI = skeletal muscle mass index; SMM: total skeletal muscle mass; TLC: total lymphocytes count.

**Table 2:** Comparison by sex of the EORTC QLQ-C30 and EORTC QLQ-CR29 questionnaires scores.

Scores of EORTC QLQ-C30 Scales					
Item and Multi-Item QoL Scales	Female		Male		*p* *
	Mean	(SD)	Mean	(SD)	
Global health status/QoL	78.29	(±19.58)	75.38	(±19.34)	0.433
Functional Scales					
Physical functioning	84.3	(±17.63)	83.79	(±17.51)	0.879
Role functioning	88.19	(±22.53)	84.87	(±24.06)	0.458
Emotional functioning	68.22	(±25.88)	78.46	(±21.69)	**0.024**
Cognitive functioning	79.51	(±23.62)	85.89	(±16.72)	0.095
Social functioning	94.79	(±13.81)	86.41	(±22.80)	**0.026**
Symptom Scales/Items					
Fatigue	25.23	(±25.08)	23.93	(±23.67)	0.779
Nausea and vomiting	8.68	(±14.98)	6.66	(±18.11)	0.531
Pain	22.91	(±29.09)	18.71	(±26.27)	0.424
Dyspnea	13.88	(±22.63)	7.17	(±16.12)	0.068
Insomnia	24.99	(±30.36)	23.58	(±29.88)	0.806
Appetite loss	15.97	(±26.62)	11.28	(±27.18)	0.362
Constipation	24.3	(±29.76)	13.33	(±23.49)	**0.043**
Diarrhea	16.66	(±29.17)	13.84	(±23.49)	0.570
Financial difficulties	32.63	(±37.34)	28.20	(±32.93)	0.505
**Scores of EORTC QLQ-CR29 Scales**					
Functional Scales					
Body Image	82.87	(±25.20)	90.42	(±15.45)	0.051
Anxiety	64.58	(±35.33)	70.76	(±32.54)	0.338
Weight	72.91	(±31.99)	80	(±29.93)	0.230
Sexual interest (men)	NA	NA	81.54	(±54.02)	NA
Sexual interest (women)	77.08	(±33.08)	NA	NA	NA
Symptom Scales/Items					
Urinary frequency	22.91	(±23.72)	21.79	(±22.03)	0.796
Blood and mucus in stool	10.76	(±16.66)	7.69	(±13.84)	0.287
Stool frequency	23.95	(±27.05)	13.84	(±19.44)	**0.023**
Urinary incontinence	5.55	(±14.31)	6.15	(±13.03)	0.818
Dysuria	9.72	(±18.13)	6.15	(±14.3)	0.245
Abdominal pain	25.69	(±29.36)	12.30	(±23.25)	**0.008**
Buttock pain	20.83	(±29.67)	12.82	(±21.80)	0.101
Bloating	30.55	(±34.26)	13.84	(±19.44)	**0.001**
Dry mouth	15.27	(±28.31)	16.40	(±25.08)	0.823
Hair loss	21.52	(±31.12)	4.61	(±13.01)	**0.000**
Taste	16.66	(±26.63)	13.84	(±25.61)	0.571
Flatulence	26.38	(±29.93)	30.25	(±32.12)	0.516
Fecal incontinence	17.36	(±31.5)	17.43	(±28.32)	0.99
Sore skin	19.44	(±29.84)	14.87	(±24.31)	0.372
Embarrassment	12.49	(±28.03)	10.76	(±25.07)	0.731
Stoma care problems	19.99	(±33.15)	8.6	(±17.714)	0.113
Impotence	NA	NA	30.25	(±37.13)	NA
Dyspareunia	13.88	(±27.36)	NA	NA	NA

* Significance level: *p* < 0.05. Statistic: Student *t*-test; EORTC QLQ-C30: European Organisation for Research and Treatment of Cancer, Quality of Life Questionnaire C30; EORTC QLQ CR29: European Organisation for Research and Treatment of Cancer Quality of Life Questionnaire specific module for Colorectal Cancer, QoL = Quality of Life; SD = standard deviation; NA = not available.

**Table 3:** Correlation of the EORTC QLQ-C30 and EORTC QLQ-CR29 questionnaires’ scores with biochemical, anthropometrical, and functional indicators of nutritional status.

Item and Multi-Item QoL Scales	Total Cholesterol rP (*p*)	Albumin rP (*p*)	Total Lymphocytes Count rP (*p*)	BMI rP (*p*)	% Fat Total rP (*p*)	% Fat Trunk rP (*p*)	Total SMM rP (*p*)	Trunk SMM rP (*p*)	Hand Grip Strength rP (*p*)
**Females**									
**Correlation of EORTC QLQ-C30 scales’ scores and body composition measures.**									
Global health status/QoL	−0.190 (0.314)	0.156 (0.394)	***0.386*** ***(0.018)***	−0.143 (0.353)	−0.299 (0.096)	***−0.349*** ***(0.05)***	−0.167 (0.361)	−0.170 (0.352)	0.250 (0.067)
Functional scales									
Physical functioning	0.150 (0.428)	0.051 (0.780)	−0.038 (0.822)	0.082 (0.596)	−0.027 (0.882)	−0.221 (0.225)	0.092 (0.616)	0.138 (0.452)	0.204 (0.263)
Role functioning	0.183 (0.334)	−0.012 (0.948)	0.132 (0.435)	0.053 (0.730)	0.056 (0.762)	−0.209 (0.251)	−0.098 (0.592)	−0.033 (0.857)	***0.399*** ***(0.024)***
Emotional functioning	−0.014 (0.941)	0.075 (0.682)	0.018 (0.917)	−0.092 (0.552)	−0.012 (0.948)	−0.091 (0.619)	0.074 (0.688)	0.077 (0.677)	0.154 (0.399)
Cognitive functioning	0.208 (0.269)	−0.212 (0.243)	0.058 (0.732)	−0.023 (0.884)	−0.084 (0.646)	−0.154 (0.401)	0.082 (0.655)	0.101 (0.581)	−0.232 (0.202)
Social functioning	0.203 (0.281)	−0.174 (0.340)	0.040 (0.815)	0.043 (0.784)	−0.001 (0.995)	−0.134 (0.463)	−0.173 (0.343)	−0.097 (0.598)	−0.085 (0.643)
Symptom scales/items									
Fatigue	0.016 (0.932)	0.027 (0.884)	−0.077 (0.652)	0.069 (0.656)	0.052 (0.776)	0.259 (0.153)	0.004 (0.982)	−0.061 (0.740)	***−0.377*** ***(0.033)***
Nausea and vomiting	−0.052 (0.787)	−0.041 (0.822)	−0.114 (0.500)	−0.002 (0.991)	−0.081 (0.660)	0.097 (0.598)	−0.031 (0.867)	−0.038 (0.835)	−0.282 (0.118)
Pain	0.112 (0.556)	−0.135 (0.463)	−0.096 (0.571)	−0.208 (0.175)	−0.174 (0.342)	−0.060 (0.745)	−0.236 (0.194)	−0.228 (0.209)	−0.312 (0.082)
Dyspnea	−0.188 (0.319)	0.114 (0.533)	−0.048 (0.776)	***0.353*** ***(0.019)***	***0.385*** ***(0.029)***	***0.484*** ***(0.005)***	***0.441*** ***(0.011)***	***0.372*** ***(0.036)***	−0.324 (0.071)
Insomnia	0.258 (0.168)	0.011 (0.953)	−0.009 (0.960)	***0.308*** ***(0.042)***	***0.372*** ***(0.036)***	0.290 (0.108)	0.130 (0.480)	0.161 (0.378)	−0.127 (0.489)
Appetite loss	0.138 (0.467)	−0.123 (0.502)	−0.020 (0.907)	−0.209 (0.858)	0.044 (0.811)	0.171 (0.349)	0.029 (0.876)	0.050 (0.787)	−0.267 (0.140)
Constipation	0.263 (0.160)	−0.161 (0.379)	0.106 (0.532)	−0.017 (0.805)	0.022 (0.904)	−0.024 (0.895)	−0.102 (0.579)	−0.102 (0.578)	0.185 (0.312)
Diarrhea	−0.166 (0.382)	0.013 (0.945)	0.023 (0.892)	−0.091 (0.557)	−0.014 (0.941)	0.176 (0.336)	0.034 (0.853)	−0.016 (0.931)	−0.052 (0.777)
Financial difficulties	−0.076 (0.688)	−0.049 (0.791)	−0.180 (0.286)	−0.074 (0.633)	0.120 (0.512)	0.218 (0.232)	−0.022 (0.904)	−0.060 (0.744)	0.078 (0.670)
**Correlation of EORTC QLQ CR 29 scales’ scores and body composition measures.**									
Functional scales									
Body Image	−0.103 (0.589)	−0.043 (0.814)	0.092 (0.589)	−0.195 (0.205)	−0.122 (0.507)	−0.244 (0.178)	−0.129 (0.482)	−0.122 (0.507)	0.247 (0.173)
Anxiety	0.018 (0.923)	−0.321 (0.073)	0.059 (0.727)	−0.087 (0.575)	0.044 (0.810)	−0.061 (0.742)	0.025 (0.893)	0.033 (0.859)	0.167 (0.361)
Weight	***0.392*** ***(0.032)***	−0.092 (0.618)	−0.204 (0.227)	−0.153 (0.320)	−0.116 (0.526)	−0.280 (0.121)	−0.125 (0.497)	−0.075 (0.682)	−0.066 (0.720)
Sexual interest (men)	0.072 (0.707)	−0.095 (0.606)	0.083 (0.626)	−0.158 (0.306)	−0.104 (0.570)	−0.151 (0.410)	−0.309 (0.085)	−0.296 (0.100)	−0.120 (0.513)
Symptom/Items Scales EORTC QLQ−CR29									
Urinary frequency	0.044 (0.819)	−0.172 (0.245)	0.195 (0.248)	0.276 (0.069)	0.218 (0.230)	0.188 (0.302)	0.252 (0.165)	0.257 (0.156)	−0.234 (0.197)
Blood and mucus in stool	***0.391*** ***(0.033)***	0.106 (0.562)	−0.189 (0.262)	0.002 (0.987)	0.016 (0.930)	0.116 (0.529)	0.053 (0.775)	0.036 (0.845)	−0.299 (0.097)
Stool frequency	−0.170 (0.370)	0.121 (0.509)	0.085 (0.617)	0.219 (0.153)	0.006 (0.974)	0.127 (0.289)	0.213 (0.243)	0.228 (0.210)	−0.246 (0.176)
Urinary incontinence	0.205 (0.276)	−0.188 (0.304)	−0.047 (0.783)	−0.123 (0.427)	0.212 (0.243)	0.235 (0.195)	0.100 (0.587)	0.085 (0.644)	−0.099 (0.588)
Dysuria	−0.104 (0.583)	0.060 (0.743)	0.009 (0.958)	−0.077 (0.618)	0.132 (0.473)	0.105 (0.566)	−0.180 (0.324)	−0.161 (0.397)	0.040 (0.828)
Abdominal pain	−0.032 (0.866)	0.068 (0.712)	0.066 (0.699)	0.061 (0.694)	−0.061 (0.739)	0.022 (0.904)	0.022 (0.903)	0.028 (0.878)	−0.149 (0.415)
Buttock pain	0.018 (0.925)	0.078 (0.673)	0.022 (0.899)	−0.047 (0.761)	−0.217 (0.233)	−0.151 (0.408)	−0.011 (0.954)	−0.036 (0.846)	−0.211 (0.247)
Bloating	0.137 (0.469)	−0.100 (0.588)	0.217 (0.197)	0.215 (0.161)	0.239 (0.187)	0.278 (0.124)	0.281 (0.120)	0.258 (0.154)	−0.275 (0.128)
Dry mouth	−0.098 (0.605)	0.043 (0.815)	0.185 (0.174)	0.189 (0.220)	0.183 (0.317)	***0.365*** ***(0.040)***	0.068 (0.713)	0.175 (0.337)	0.009 (0.963)
Hair loss	−0.017 (0.929)	0.147 (0.422)	−0.139 (0.411)	−0.204 (0.184)	−0.307 (0.088)	−0.268 (0.138)	−0.288 (0.110)	−0.340 (0.057)	0.129 (0.483)
Taste	0.131 (0.490)	−0.143 (0.436)	−0.113 (0.504)	−0.185 (0.230)	−0.007 (0.968)	0.124 (0.498)	−0.007 (0.970)	−0.033 (0.857)	−0.246 (0.174)
Flatulence	0.097 (0.611)	0.062 (0.737)	0.184 (0.274)	−0.022 (0.887)	−0.172 (0.346)	−0.126 (0.492)	−0.310 (0.078)	−0.292 (0.104)	0.057 (0.756)
Fecal incontinence	0.140 (0.462)	0.087 (0.635)	0.003 (0.984)	***0.305*** ***(0.044)***	0.175 (0.339)	0.159 (0.383)	0.195 (0.284)	0.141 (0.441)	−0.304 (0.090)
Sore skin	0.073 (0.703)	−0.010 (0.959)	0.069 (0.687)	−0.018 (0.907)	−0.158 (0.388)	−0.095 (0.603)	−0.082 (0.654)	−0.120 (0.512)	0.173 (0.344)
Embarrassment	−0.105 (0.580)	0.065 (0.724)	−0.071 (0.678)	0.295 (0.052)	0.238 (0.189)	***0.388*** ***(0.028)***	0.157 (0.390)	0.243 (0.179)	***−0.367*** ***(0.039)***
Stoma care problems	0.011 (0.971)	0.237 (0.395)	−0.175 (0.517)	−0.085 (0.746)	−0.182 (0.534)	0.191 (0.513)	−0.068 (0.818)	−0.167 (0.567)	−0.211 (0.451)
Dyspareunia	−0.085 (0.654)	0.049 (0.791)	−0.010 (0.955)	−0.108 (0.485)	−0.084 (0.649)	−0.063 (0.731)	−0.110 (0.547)	−0.085 (0.643)	***0.495*** ***(0.004)***
**Males**									
**Correlation of EORTC QLQ-C30 scales’ scores and body composition measures.**									
Global health status/QoL	−0.008 (0.962)	0.070 (0.673)	0.123 (0.367)	−0.079 (0.572)	***−0.347*** ***(0.041)***	***−0.341*** ***(0.045)***	0.251 (0.145)	***0.506*** ***(0.002)***	0.161 (0.356)
Functional scales									
Physical functioning	0.143 (0.413)	***0.395*** ***(0.013)***	0.001 (0.994)	−0.030 (0.827)	−0.005 (0.978)	−0.159 (0.361)	0.117 (0.504)	***0.560*** ***(0.000)***	***0.337*** ***(0.048)***
Role functioning	0.094 (0.589)	0.264 (0.104)	0.051 (0.709)	−0.064 (0.643)	−0.001 (0.997)	−0.080 (0.650)	0.040 (0.819)	***0.580*** ***(0.000)***	0.329 (0.053)
Emotional functioning	0.254 (0.140)	***0.453*** ***(0.004)***	−0.125 (0.359)	0.162 (0.240)	0.080 (0.646)	−0.059 (0.738)	***0.450*** ***(0.011)***	***0.567*** ***(0.000)***	***0.324*** ***(0.057)***
Cognitive functioning	0.080 (0.647)	0.037 (0.823)	−0.117 (0.389)	0.133 (0.338)	−0.006 (0.971)	0.035 (0.842)	0.297 (0.083)	0.007 (0.966)	0.111 (0.526)
Social functioning	0.127 (0.468)	***0.363*** ***(0.023)***	0.158 (0.245)	−0.007 (0.958)	0.066 (0.706)	−0.016 (0.927)	0.255 (0.140)	***0.625*** ***(0.000)***	***0.347*** ***(0.041)***
Symptom scales/items									
Fatigue	0.179 (0.302)	***−0.36*** ***(0.024)***	0.112 (0.411)	−0.036 (0.794)	−0.067 (0.702)	−0.014 (0.937)	0.267 (0.122)	***−0.586*** ***(0.000)***	***−0.376*** ***0.026)***
Nausea and vomiting	−0.144 (0.409)	***−0.387*** ***(0.015)***	0.014 (0.920)	−0.034 (0.807)	−0.123 (0.480)	−0.049 (0.778)	−0.092 (0.598)	***−0.469*** ***(0.005)***	−0.216 (0.212)
Pain	0.083 (0.635)	−0.262 (0.108)	0.008 (0.951)	0.047 (0.735)	−0.004 (0.980)	0.034 (0.847)	−0.142 (0.416)	***0.506*** ***(0.002)***	−0.307 (0.073)
Dyspnea	0.312 (0.068)	−0.177 (0.281)	0.170 (0.212)	−0.200 (0.147)	−0.148 (0.396)	0.065 (0.712)	−0.135 (0.439)	−0.298 (0.082)	−0.058 (0.741)
Insomnia	−0.136 (0.437)	0.027 (0.869)	−0.053 (0.697)	−0.022 (0.875)	0.028 (0.874)	−0.027 (0.876)	−0.289 (0.092)	−0.028 (0.872)	0.053 (0.762)
Appetite loss	−0.228 (0.188)	***−0.347*** ***(0.030)***	0.137 (0.315)	−0.195 (0.158)	−0.327 (0.056)	−0.160 (0.360)	−0.105 (0.547)	−0.244 (0.158)	−0.180 (0.301)
Constipation	0.077 (0.658)	***−0.331*** ***(0.039)***	0.148 (0.277)	−0.013 (0.028)	−0.150 (0.289)	−0.165 (0.345)	−0.099 (0.570)	−0.077 (0.659)	***−0.468*** ***(0.005)***
Diarrhea	−0.322 (0.060)	***−0.455*** ***(0.004)***	−0.144 (0.291)	−0.114 (0.413)	−0.170 (0.329)	−0.005 (0.978)	−0.028 (0.872)	−0.322 (0.059)	−0.187 (0.286)
Financial difficulties	−0.073 (0.677)	−0.290 (0.073)	−0.152 (0.263)	0.142 (0.305)	0.125 (0.473)	0.105 (0.548(	−0.216 (0.213)	***−0.475*** ***(0.004)***	−0.135 (0.439)
**Correlation of EORTC QLQ-CR29 scales’ scores and body composition measures.**									
Functional scales									
Body Image	0.162 (0.353)	***0.372*** ***(0.020)***	−0.002 (0.990)	0.019 (0.893)	0.106 (0.545)	0.013 (0.939)	0.226 (0.191)	***0.448*** ***(0.007)***	0.296 (0.085)
Anxiety	0.329 (0.054)	0.034 (0.839)	0.073 (0.595)	0.044 (0.749)	0.007 (0.967)	−0.063 (0.719)	0.318 (0.063)	***0.505*** ***(0.002)***	0.267 (0.121)
Weight	0.251 (0.145)	***0.404*** ***(0.011)***	0.006 (0.962)	0.100 (0.470)	0.126 (0.471)	0.056 (0.748)	0.116 (0.508)	***0.468*** ***(0.005)***	0.302 (0.078)
Sexual interest (men)	−0.057 (0.746)	0.016 (0.925)	−0.041 (0.765)	−0.226 (0.100)	−0.296 (0.085)	−0.142 (0.415)	0.015 (0.931)	0.019 (0.912)	−0.278 (0.106)
Symptom/Items Scales									
Urinary frequency	0.157 (0.368)	0.149 (0.365)	0.056 (0.680)	−0.073 (0.600)	0.042 (0.809)	0.084 (0.629)	−0.204 (0.240)	***−0.365*** ***(0.031)***	−0.158 (0.365)
Blood and mucus in stool	−0.008 (0.964)	0.088 (0.596)	0.073 (0.594)	−0.263 (0.055)	−0.104 (0.552)	−0.181 (0.297)	−0.224 (0.196)	−0.002 (0.991)	−0.061 (0.728)
Stool frequency	0.026 (0.881)	−0.105 (0.523)	0.099 (0.467)	−0.092 (0.509)	−0.145 (0.405)	−0.027 (0.880)	0.074 (0.671)	0.077 (0.661)	0.045 (0.796)
Urinary incontinence	−0.212 (0.221)	−0.302 (0.062)	−0.099 (0.466)	−0.050 (0.721)	−0.084 (0.633)	0.012 (0.948)	0.054 (0.759)	0.009 (0.958)	***−0.363*** ***(0.032)***
Dysuria	0.092 (0.597)	−0.121 (0.464)	−0.065 (0.634)	0.001 (0.995)	−0.217 (0.210)	−0.119 (0.496)	0.124 (0.479)	0.050 (0.775)	−0.066 (0.704)
Abdominal pain	−0.225 (0.193)	−0.281 (0.083)	0.054 (0.692)	−0.151 (0.277)	−0.315 (0.065)	−0.311 (0.069)	−0.253 (0.143)	−0.108 (0.537)	−0.272 (0.114)
Buttock pain	0.048 (0.785)	−0.218 (0.182)	0.086 (0.527)	0.193 (0.162)	0.106 (0.545)	0.159 (0.361)	−0.016 (0.929)	−0.293 (0.088)	−0.097 (0.578)
Bloating	−0.140 (0.423)	−0.237 (0.146)	−0.036 (0.793)	−0.182 (0.188)	−0.270 (0.116)	−0.244 (0.159)	0.050 (0.777)	−0.105 (0.547)	−0.150 (0.389)
Dry mouth	−0.192 (0.268)	−0.193 (0.240)	−0.106 (0.439)	−0.181 (0.190)	−0.278 (0.106)	−0.139 (0.427)	−0.081 (0.644)	−0.294 (0.087)	−0.206 (0.234)
Hair loss	−0.270 (0.117)	0.119 (0.469)	−0.043 (0.756)	0.092 (0.509)	0.208 (0.230)	0.114 (0.515)	−0.197 (0.257)	−0.017 (0.922)	−0.097 (0.580)
Taste	0.081 (0.643)	−0.043 (0.796)	−0.016 (0.906)	***0.321*** ***(0.018)***	***0.421*** ***(0.012)***	0.287 (0.095)	−0.025 (0.888)	***−0.497*** ***(0.004)***	0.026 (0.883)
Flatulence	−0.290 (0.090)	−0.137 (0.406)	−0.055 (0.689)	0.163 (0.238)	0.154 (0.377)	0.066 (0.706)	−0.202 (0.245)	−0.146 (0.403)	***−0.394*** ***(0.019)***
Fecal incontinence	0.102 (0.561)	−0.188 (0.253)	0.103 (0.452)	−0.042 (0.761)	−0.042 (0.811)	0.031 (0.858)	−0.162 (0.353)	−0.153 (0.379)	−0.315 (0.065)
Sore skin	0.027 (0.878)	−0.148 (0.370)	−0.167 (0.219)	−0.005 (0.969)	−0.238 (0.169)	−0.231 (0.182)	−0.114 (0.515)	−0.006 (0.971)	−0.192 (0.270)
Embarrassment	−0.059 (0.738)	−0.302 (0.062)	0.066 (0.628)	0.000 (1.000)	−0.069 (0.694)	0.055 (0.752)	−0.087 (0.617)	−0.089 (0.609)	***−0.420*** ***(0.012)***
Stoma care problems	0.2478 (0.324)	0.110 (0.634)	0.121 (0.549)	***0.443*** ***(0.021)***	***0.738*** ***(0.000)***	***0.775*** ***(0.000)***	0.011 (0.966)	−0.589 (0.010)	0.367 (0.134)
Impotence	0.074 (0.673)	−0.258 (0.112)	−0.111 (0.414)	−0.053 (0.706)	−0.211 (0.244)	−0.094 (0.592)	0.162 (0.351)	−0.025 (0.887)	−0.064 (0.716)

BMI: body mass index; EORTC QLQ-C30: European Organisation for Research and Treatment of Cancer, Quality of Life Questionnaire C30; EORTC QLQ CR29: European Organisation for Research and Treatment of Cancer Quality of Life Questionnaire specific module for Colorectal Cancer; BMI: body mass index; SMM: skeletal muscle mass.; QoL: quality of life.
